# Supplementary material for: Molecularly-porous ultrathin membranes for highly selective organic solvent nanofiltration
Source: Nat Commun. 2020 Nov 18;11:5882. doi: 10.1038/s41467-020-19404-6 (PMC7674481; doi:10.1038/s41467-020-19404-6)
Supplement: Supplementary file 1 — Supplementary Information [file 41467_2020_19404_MOESM1_ESM.pdf]

Supporting Information

Molecularly-Porous Ultrathin Membranes for Highly Selective Organic Solvent Nanofiltration

Tiefan Huang[1, 2], Basem A. Moosa[3], Phuong Hoang[3], Jiangtao Liu[1], Stefan Chisca[1], Gengwu Zhang[3], Mram AlYami[3], Niveen M. Khashab[3*], Suzana P. Nunes[1*]

[1] King Abdullah University of Science and Technology (KAUST), Nanostructured Polymeric Membranes Laboratory, Advanced Membranes and Porous Materials Center, Biological and Environmental Science and Engineering Division (BESE), Thuwal, 23955-6900, Saudi Arabia

[2] Functional Membrane Materials Engineering Research Center of Hunan Province, School of Chemistry and Chemical Engineering, Hunan University of Science and Technology, Xiangtan 411201, China

[3] King Abdullah University of Science and Technology (KAUST), Smart Hybrid Materials (SHMs) Laboratory, Advanced Membranes and Porous Materials Center, Physical Sciences and Engineering Division (PSE), Thuwal, 23955-6900, Saudi Arabia

Molecular simulations

Molecular modelling of trianglamine was constructed by referring to the single crystal structure. Protons of amine groups were omitted due to amide reaction during membrane formation. Molecular electrostatic potential (EPS) was mapped with the Gaussian 09 software package by density functional theory (DFT) calculations at B3LYP/6-31G level.

Models of monomer molecules are constructed and described with polymer-consistent force field (PCFF). 20 trianglamine molecules and 60 TPC molecules are packed in a cubic cell with dimension of 65 Angstrom, at low density of 0.4 g/cm^3. For modeling the membrane prepared from trianglamine fragments, 60 linear molecules and 60 TPC molecules were packed in a cubic cell with dimension of 48.8 Angstrom. The chlorines in TPC molecules and the amino hydrogens in trianglamine molecules are removed. Carbons of carbonyls in TPC molecules and nitrogens in trianglamine molecules are marked with a tag, then polymerization step is performed between tagged carbons and nitrogens within a cutoff of 6 Angstrom, with energy minimization and MD steps to adjust molecules. Polymatic is used in generating amorphous polymer models and polymerization.[1,2] The polymerized molecule is performed with a 21-step equilibration.[3] Unreacted ends of carbonyls and amine are restored to chlorines and hydrogens. LAMMPS is used

in both MD step of polymerization and equilibration.[4] Material Studio is used to analyze accessible surface, with a probe of 1 Å radius. Zeo++ is used to analyze voids, including void space, pore size distribution and the interconnectivity of void space,[5] when probe radii are 0.85 Å, 1.2 Å and 1.55 Å.

XPS calculation

Because nitrogen element only comes from trianglamine, we can calculate the percentage of reacted -NH_2 in trianglamine by following equation,

$$Percentage\ of\ reacted - NH - group = {S_{O=C-N}}\Big/{S_{-NH-} + S_{-N-} + S_{O=C-N}}$$

Where $S_{O=C-N}$, S_{-NH-}, and S_{-N+-} were the peak area of O=C-N, -NH-, and -N^+- components in N1s narrow scan, respectively. The percentage of the reacted -NH2 was calculated to be 78%.

Based on the results above, we also can further calculate the crosslinking degree of the trianglamine in the film by following equations,

$$X + Y = 6 \times 0.78$$

$$\frac{Y}{X+Y} = \frac{S_{COO}}{S_{N-C=O}}$$

Where X represent the normalized number of crosslinked TPC in the film, Y the uncrosslinked TPC. X was calculated to be 4.1, and Y was calculated to be 0.6, which means that each trianglamine crosslinked with about 4 others to formed the hyper-cross-linked membrane.

Trianglamine content in the film can be calculated as follows

$$w_{trianglamine} = {W_{trianglamine}}\Big/{W_{trianglamine} + W_{TPC}}$$
$$= {M_{trianglamine}}\Big/{M_{trianglamine} + \frac{X}{2} \times M_{TPC} + Y \times M_{TPC}}$$

Where $w_{trianglamine}$ was the weight percentage of trianglamine in the film, $W_{trianglamine}$ and W_{TPC} were the weight of trianglamine and TPC, respectively in the film, $M_{trianglamine}$ and M_{TPC} were the molecular weight of trianglamine and TPC, respectively. The weight percentage of trianglamine in the film was calculated to be 60 %.

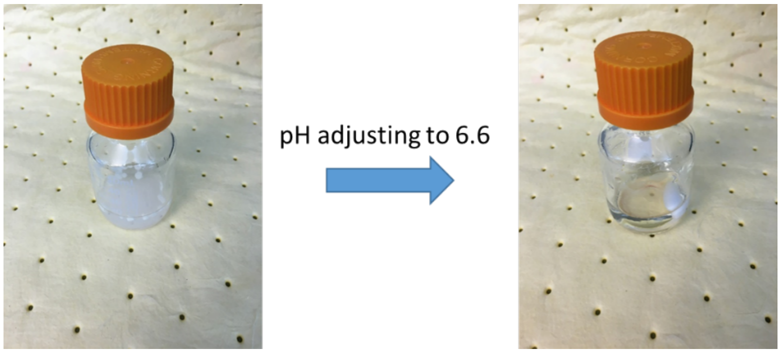

Supplementary Figure 1. Trianglamine solution before and after adjusting the pH.

Characterization by Electrospray Mass Spectroscopy (ESI-MS)

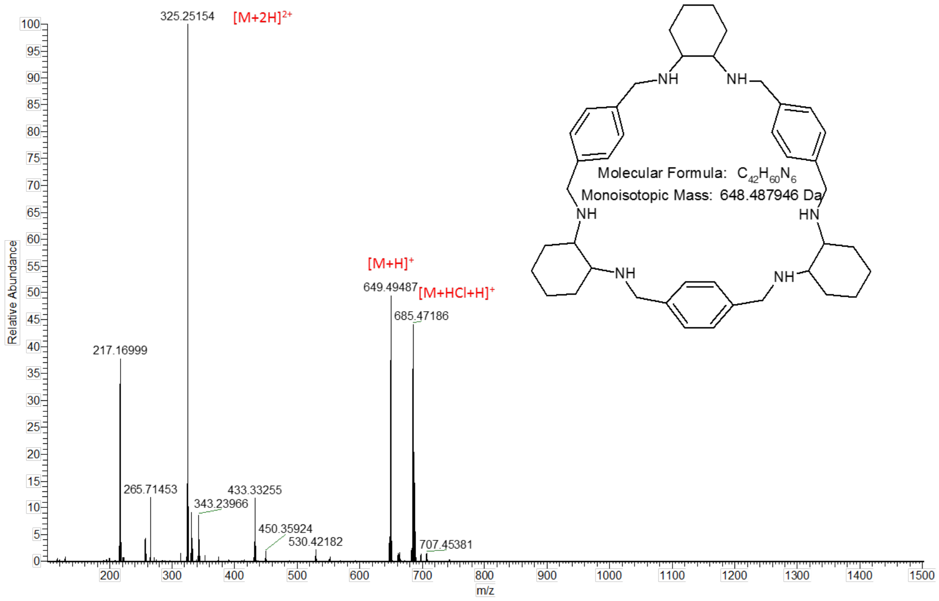

Supplementary Figure 2. ESI-MS of trianglamine solution in water under pH = 6.6.

Morphology

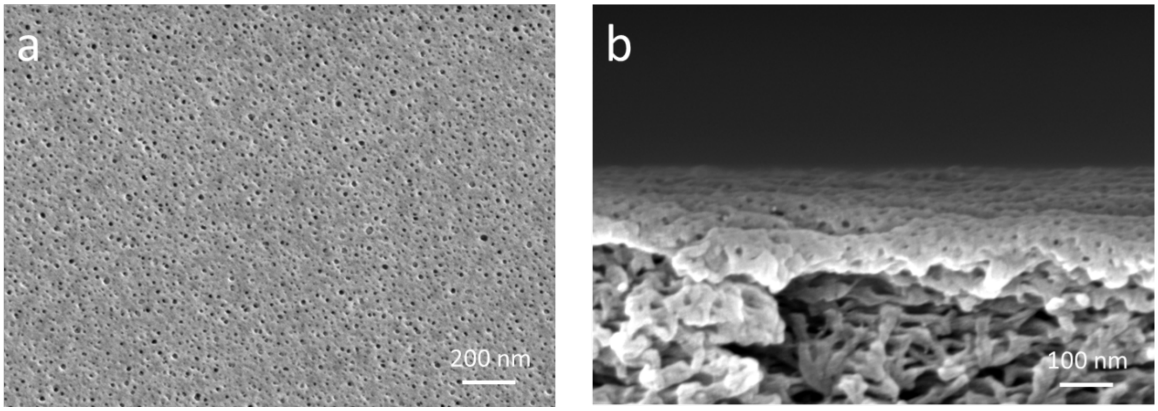

Supplementary Figure 3. SEM images of the PAN support. **a,** Surface. **b,** Cross-section.

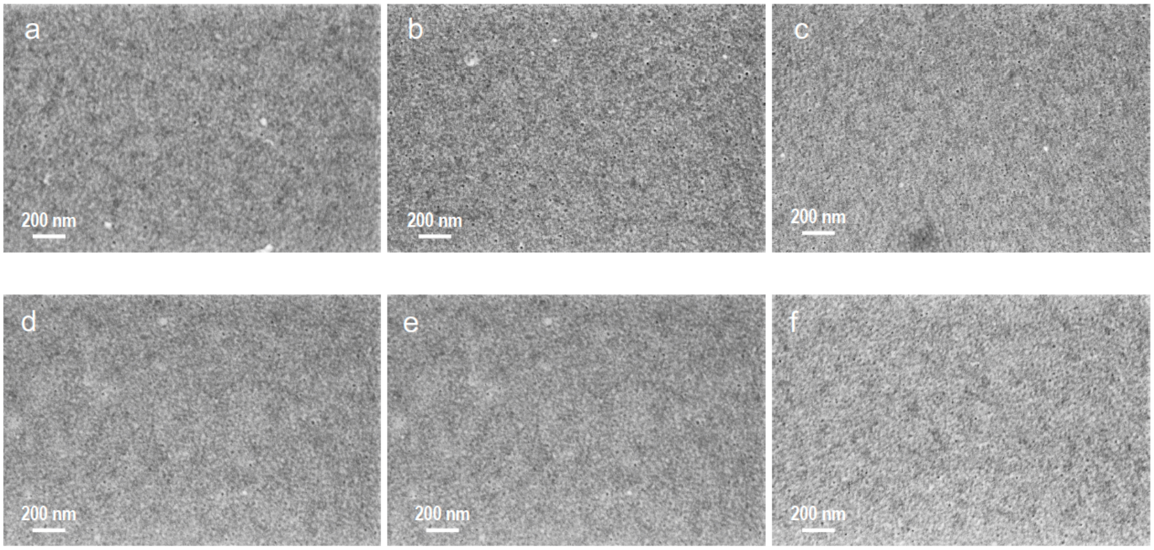

Supplementary Figure 4. SEM images of the surface of MPCM/PAN thin-film composite membranes with 2% trianglamine in the aqueous phase. Reaction times: **a**, 10min; **b**, 5min; **c**, 2min; **d**, 1min; **e**, 30s; **f**, 10s.

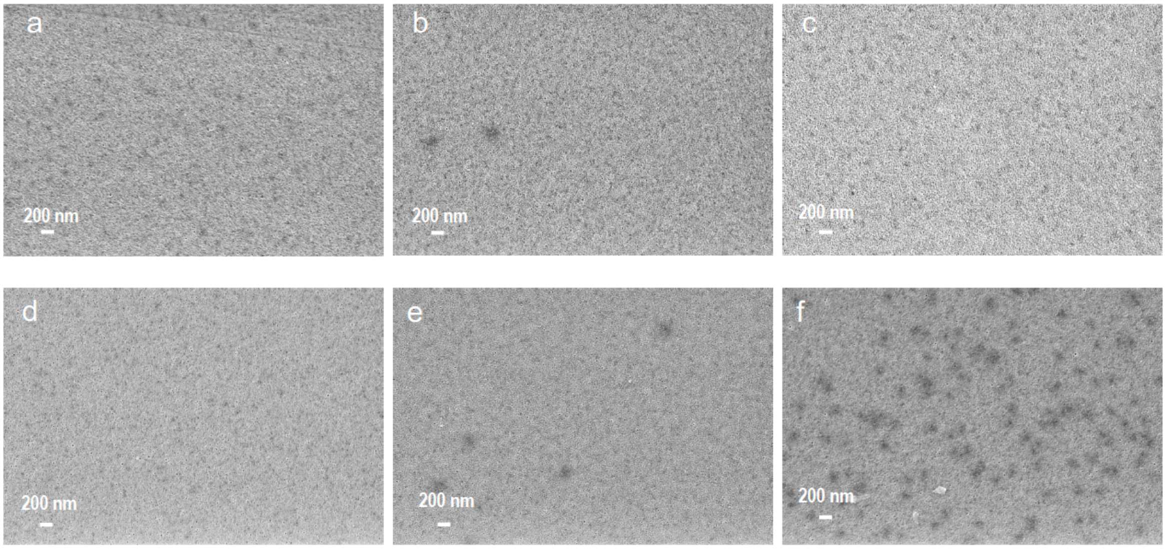

Supplementary Figure 5. SEM images of the surface of MPCM/PAN thin-film composite membranes with 1% trianglamine in the aqueous phase. Reaction times: **a**, 10min; **b**, 5min; **c**, 2min; **d**, 1min; **e**, 30s; **f**, 10s.

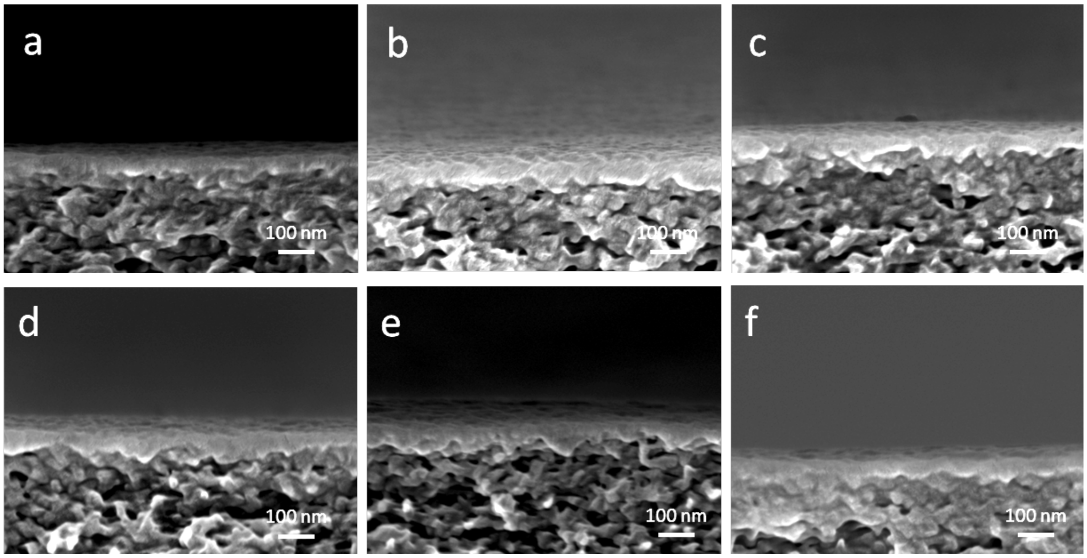

Supplementary Figure 6. SEM images of the cross-section of MPCM/PAN thin-film composite membranes with 2% trianglamine in the aqueous phase. Reaction times: **a**, 10min; **b**, 5min; **c**, 2min; **d**, 1min; **e**, 30s; **f**, 10s.

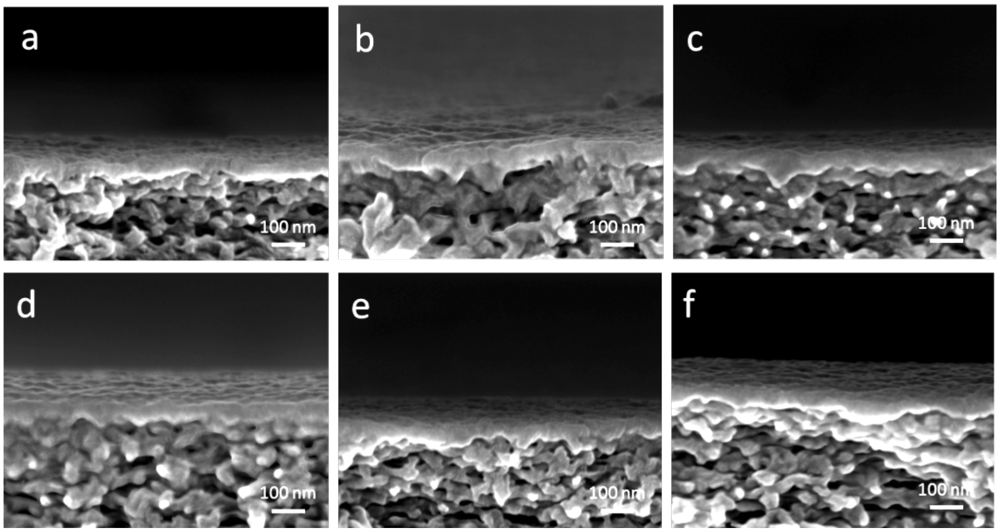

Supplementary Figure 7. SEM images of the cross-section of MPCM/PAN thin-film composite membranes with 1% trianglamine in the aqueous phase. Reaction times: **a**, 10min; **b**, 5min; **c**, 2min; **d**, 1min; **e**, 30s; **f**, 10s.

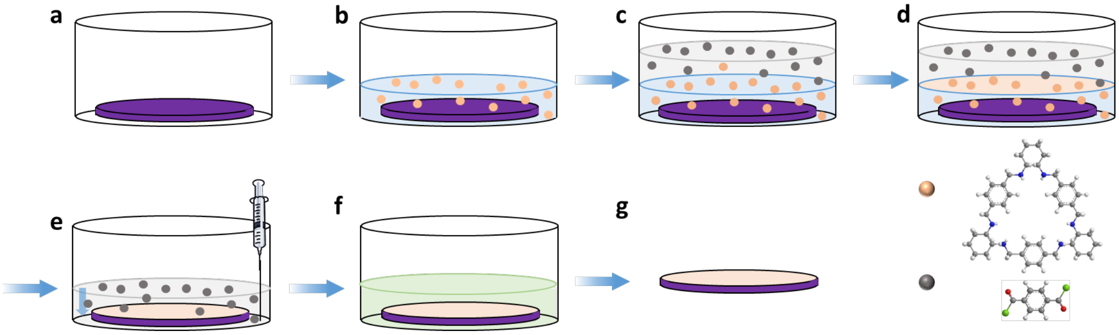

Supplementary Figure 8. Schematic of free-standing MPCM nanofilms fabricated at a free interface between organic phase and aqueous phase. **a**, Silica wafer at the bottom of petri dish. **b-d**, Instantaneous formation of the layer at the interface between an aqueous phase containing trianglamine and organic phase containing TPC. **e**, Lowing the film onto the substrate by decreasing the interface via syringe. **f**, the film was washed by hexane.

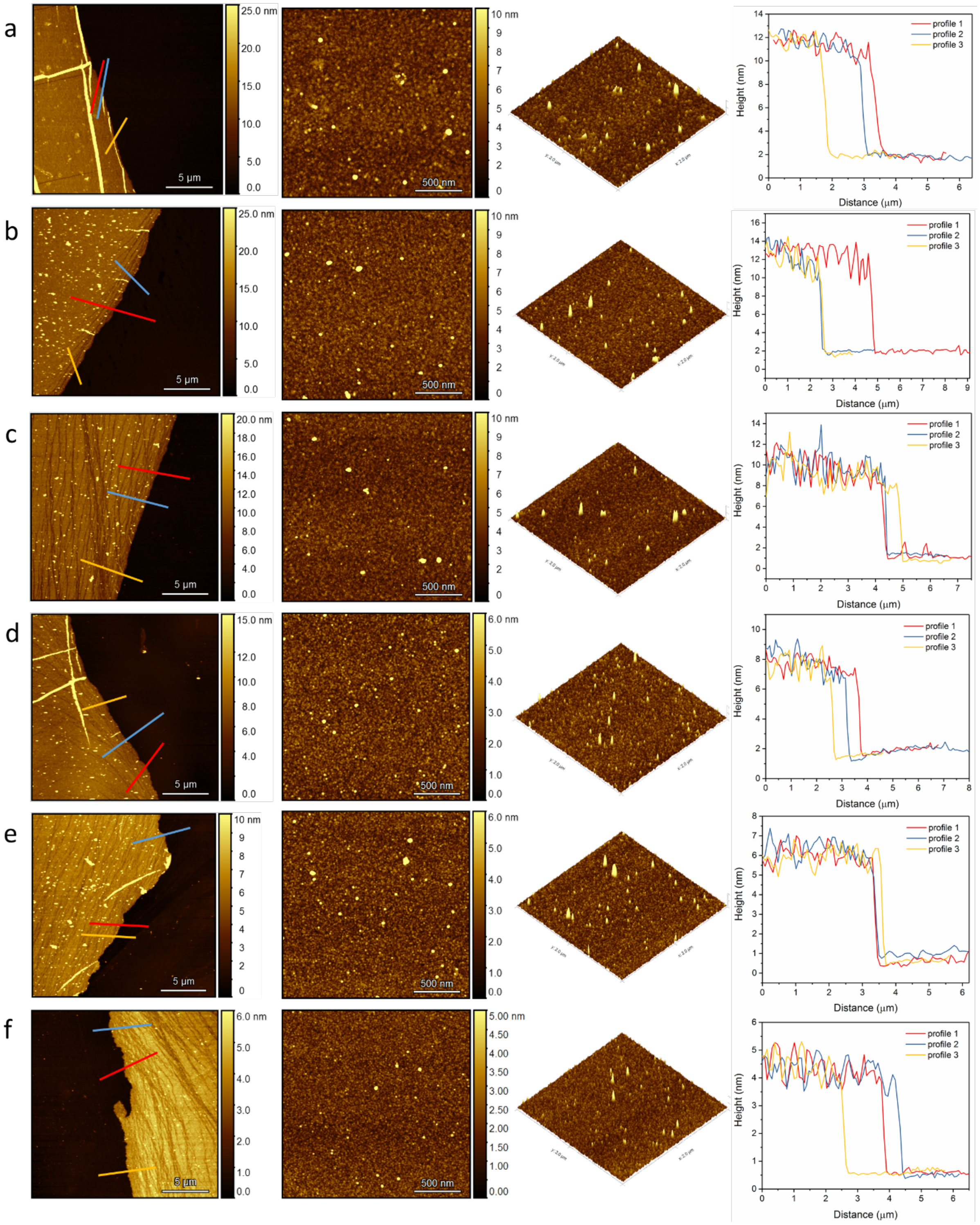

Supplementary Figure 9. AFM images (2D and 3D representation) and height profile of freestanding MPCM nanofilms, prepared with 2 % trianglamine with different reaction times: **a**, 10min **b**, 5min, **c**, 2min, **d**, 1min, **e**, 30s, and **f**, 10s.

.

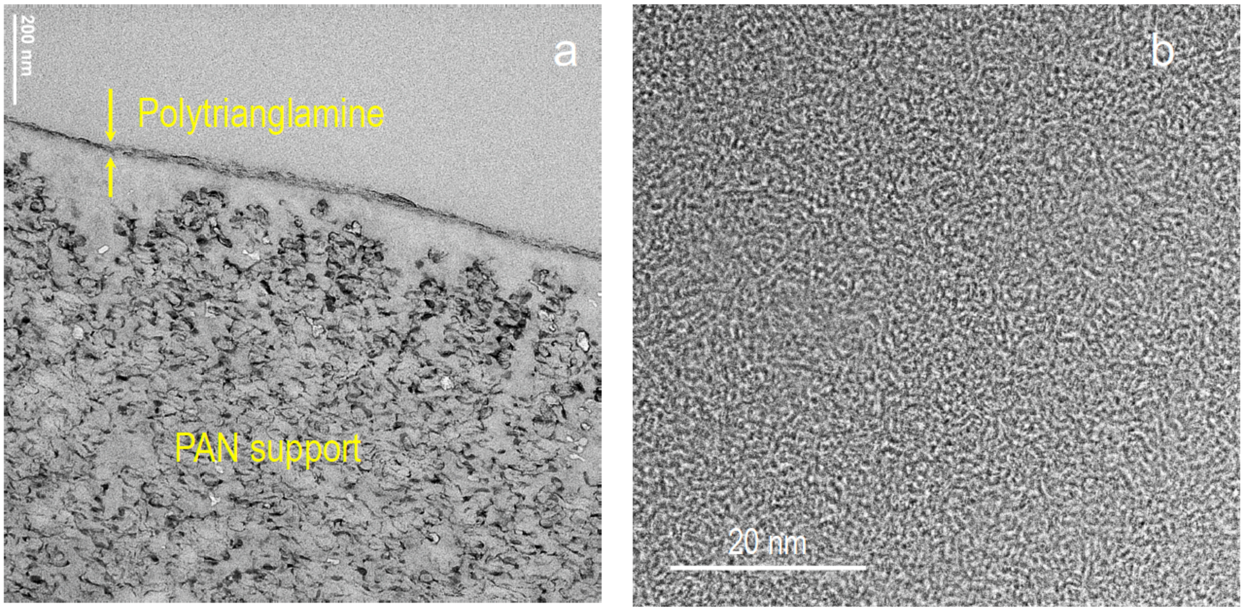

Supplementary Figure 10. High resolution TEM image of MPCM nanofilms. **a,** Cross-section of the polytrianglamine film on PAN support, prepared with 2 % trianglamine and 10 min reaction time; **b,** HR-TEM images of a freestanding polytrianglamine film. The samples were stained with ruthenium oxide.

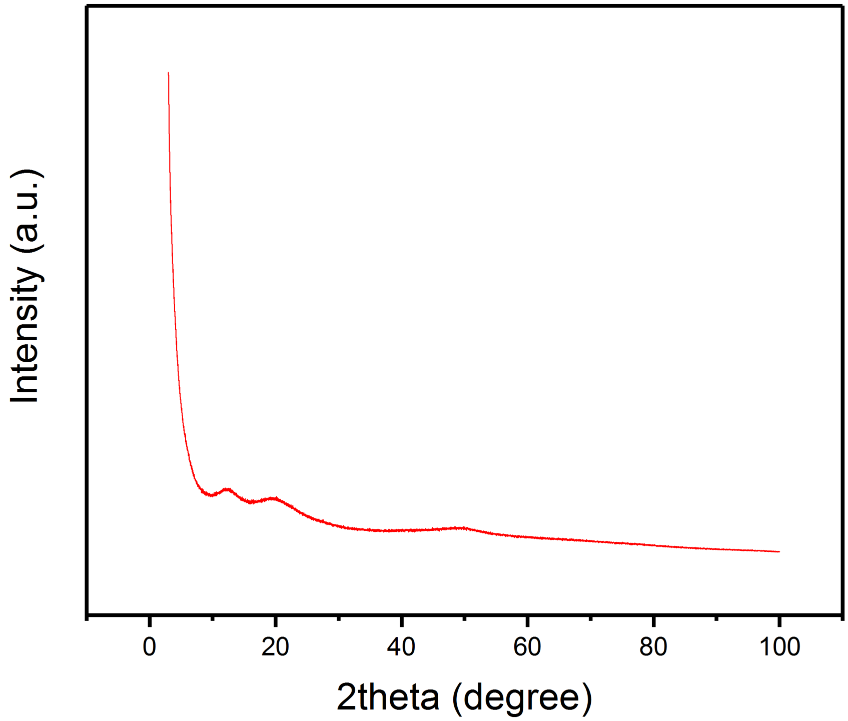

Supplementary Figure 11. XRD pattern of the MPCM nanofilms.

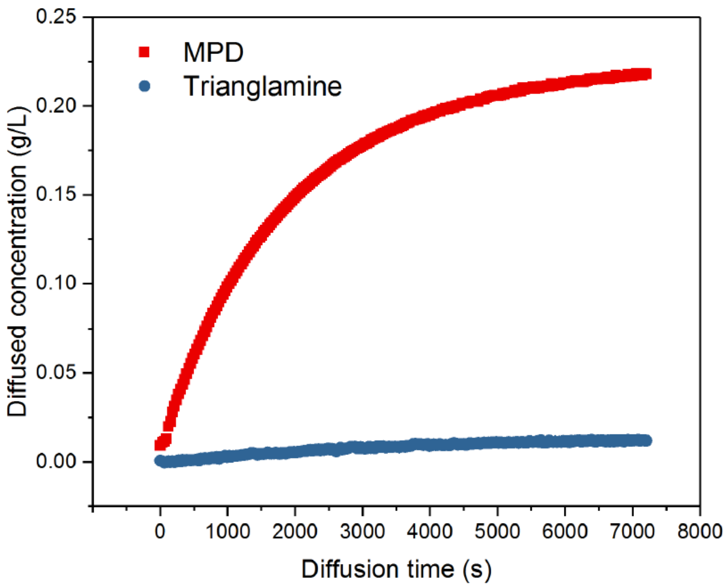

Supplementary Figure 12. Concentration of MPD (red) and trianglamine (blue) monitored at the testing point in hexane phase.

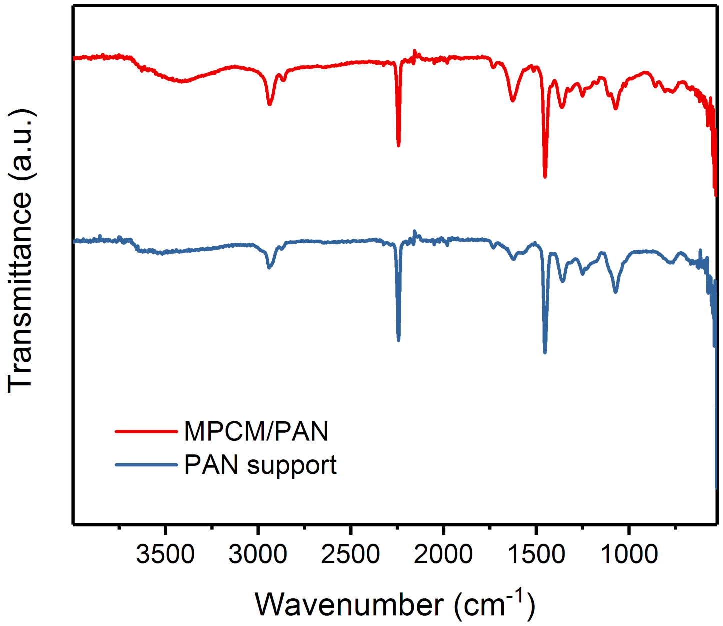

Supplementary Figure 13. ATR-FTIR spectra of PAN support (in blue) and MPCM/PAN thin-film composite membrane (in red).

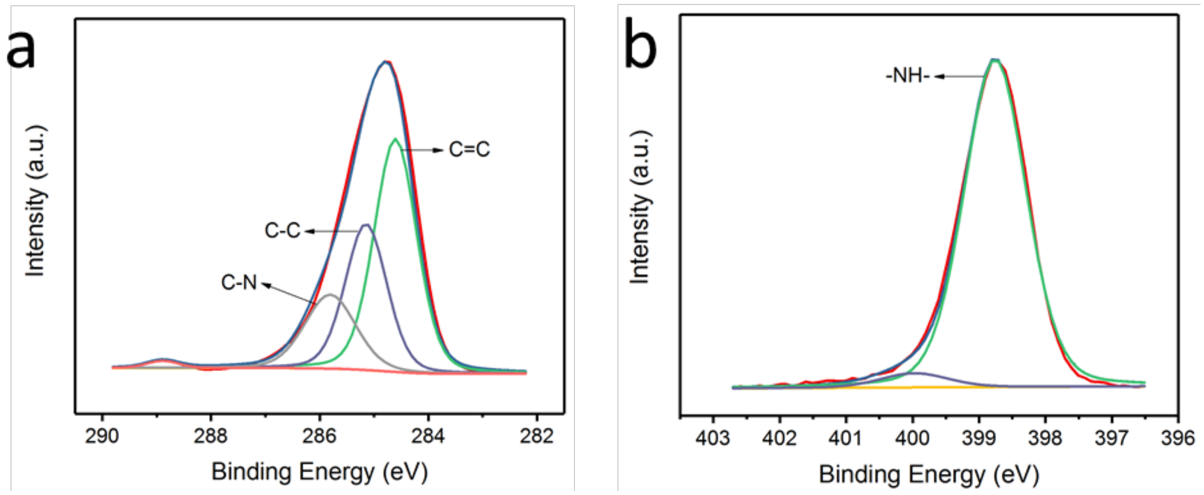

Supplementary Figure 14. XPS characterization. **a,** C1s and **b,** N1s high resolution spectra of trianglamine.

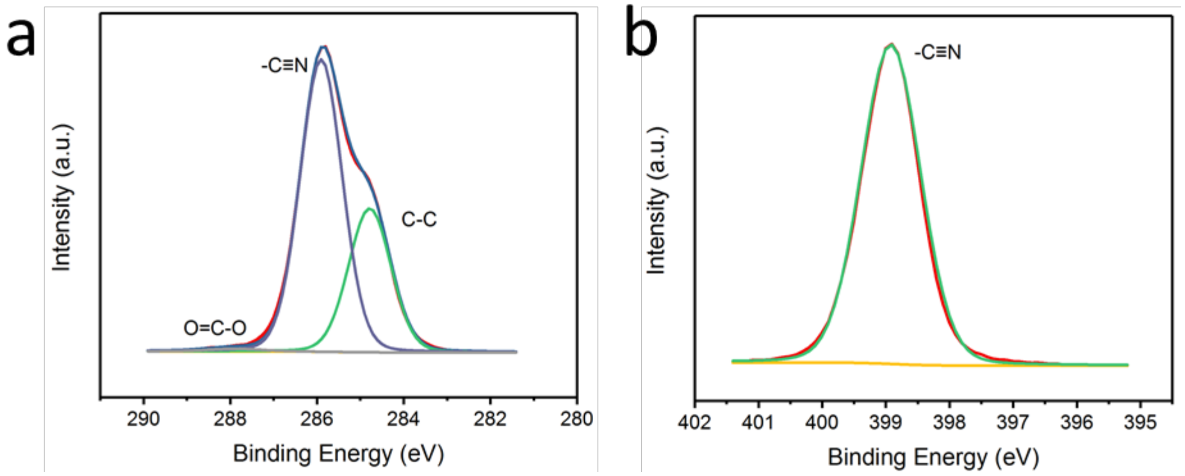

Supplementary Figure 15. XPS characterization. **a,** C1s and **b,** N1s high-resolution spectra of PAN support.

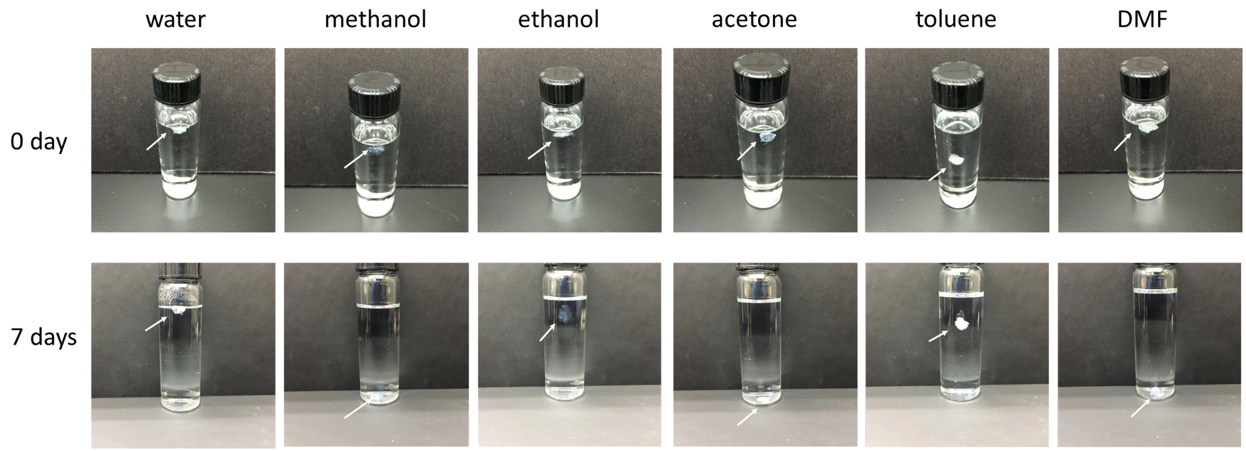

Supplementary Figure 16. As-prepared MPCM nanofilms in various organic solvents. **Top,** immediate immersion; **down,** immersion after 7 days.

Contact angle

Water contact angle (CA) was measured on a Kruss drop shaper analyzer DSA100 with a monochrome interline CCD camera. For each experiment, 5μL of water was injected and contacted with the sample surface, and the water contact angle is automatically calculated with the fitting method of Elipse (Tangent-1).

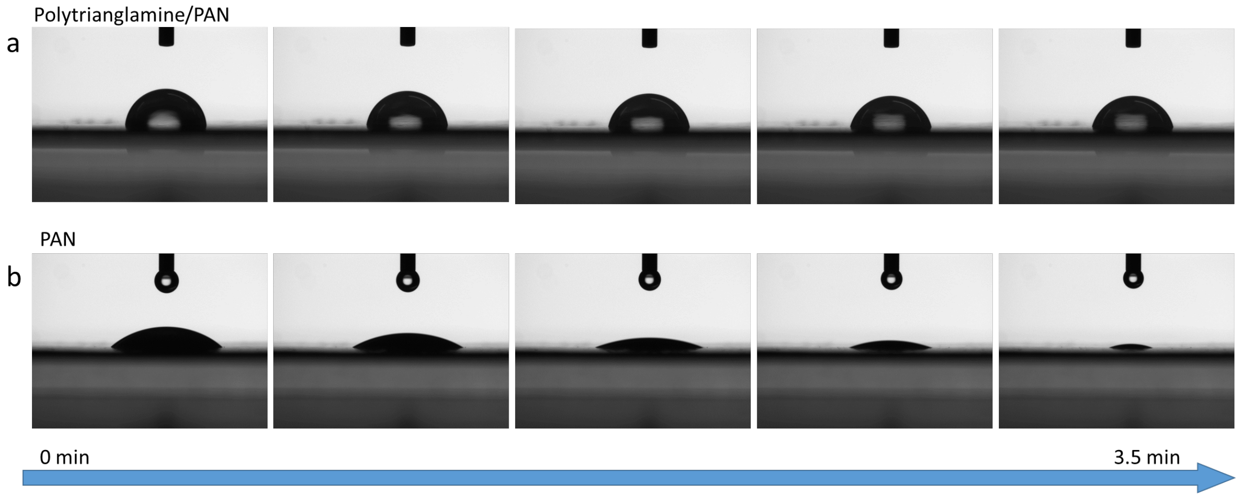

Supplementary Figure 17. Water droplets. **a,** on a MPCM/PAN thin-film composite membrane**. b,** PAN porous support**.**

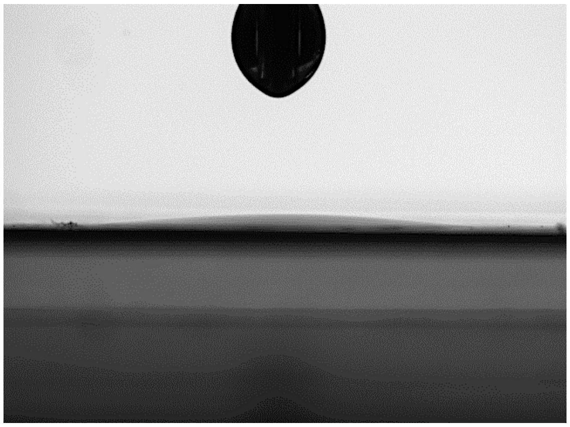

Supplementary Figure 18. Hexane contact angle on MPCM/PAN thin-film composite membrane.

Performance

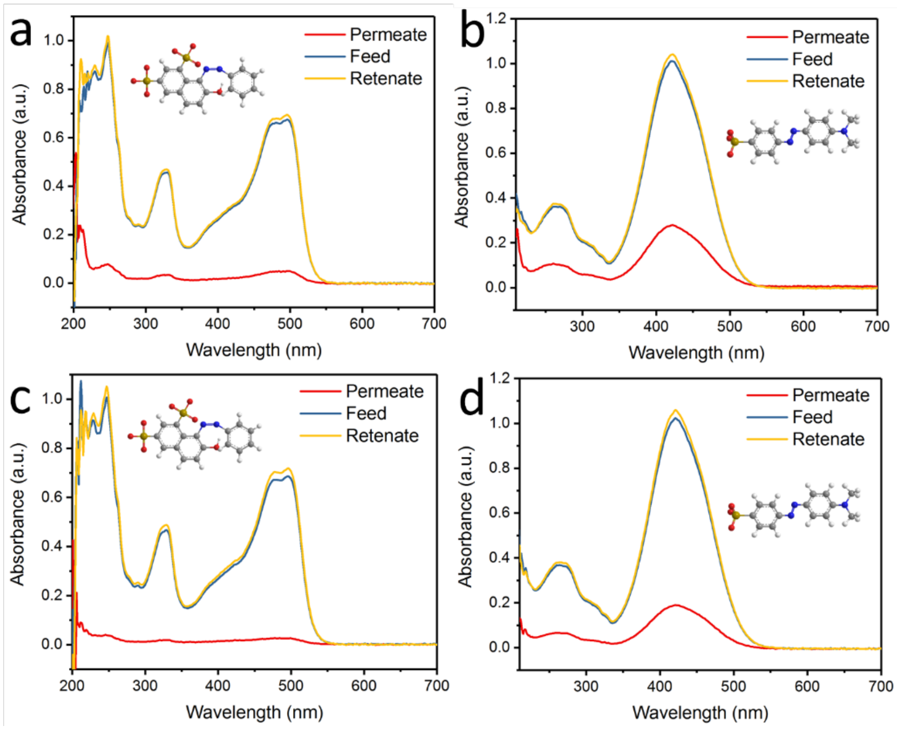

Supplementary Figure 19. Separation performance stability of MPCM/PAN thin-film composite membranes before (a, b) and after (c, d) a series of organic solvents filtrations; (a, c) Orange G and (b, d) Methyl Orange.

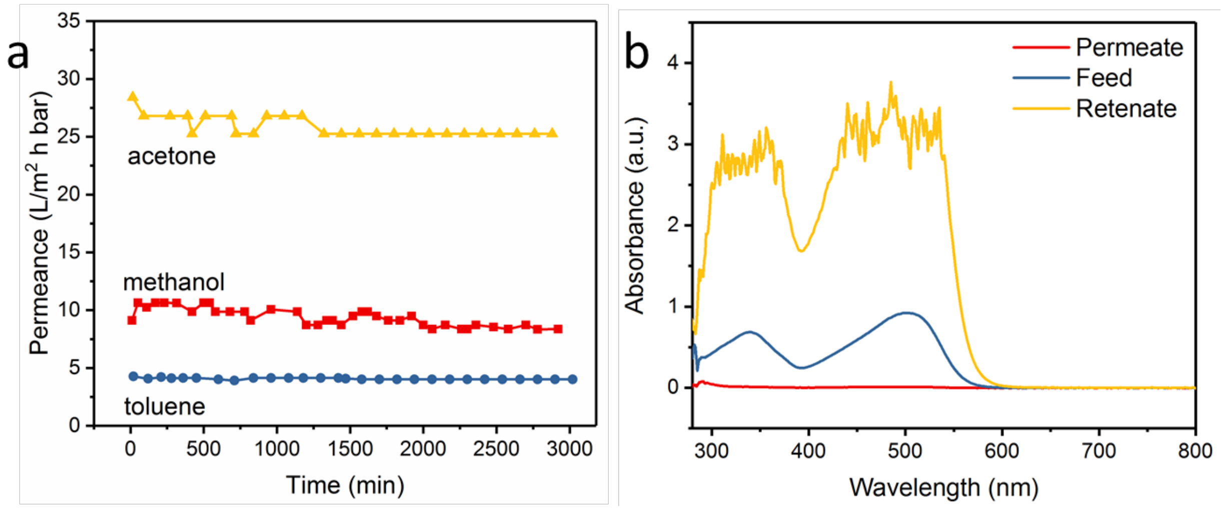

Supplementary Figure 20. The long-term stability. MPCM/PAN thin-film composite membrane prepared by 2% of aqueous phase concentration and reaction time of 10 min tested **a**, by acetone, methanol and toluene and **b**, congo red solution in methanol (100 ppm) for more than 48h.

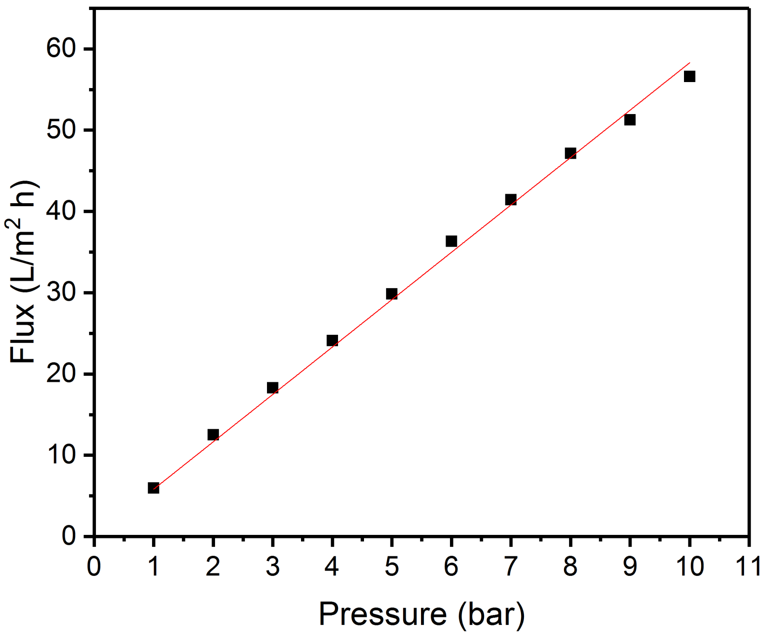

Supplementary Figure 21. Permeation vs applied pressure. Toluene permeation flux of MPCM/PAN thin-film composite membrane with reaction time of 10 min**.**

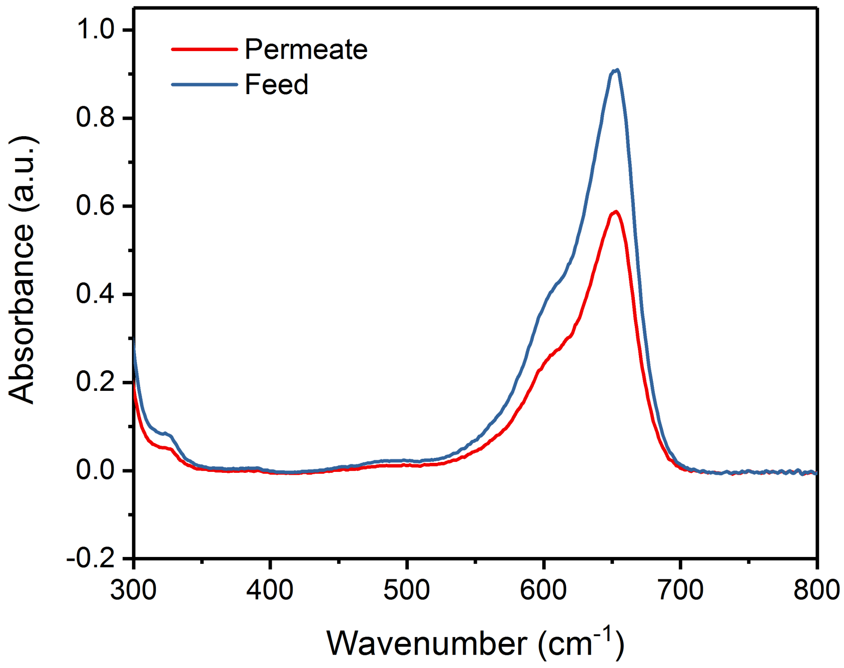

Supplementary Figure 22. Separation performance of MPCM/PAN thin-film composite membrane for positive dye methylene blue.

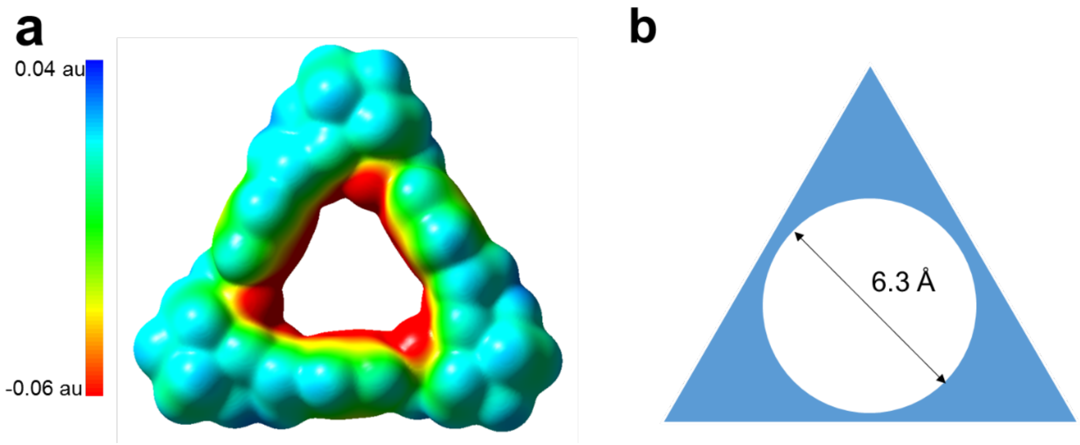

Supplementary Figure 23. a, Electrostatic potential (ESP) mapped onto electron density isosurfaces (ρ=0.01) for trianglamine**. b,** Schematic illustration for the cavity of trianglamine (cavity size value from single crystal structure).

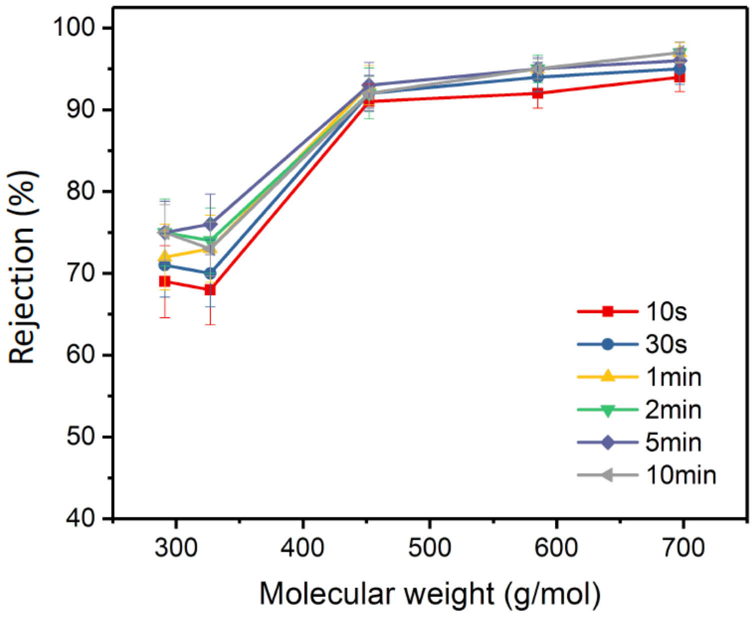

Supplementary Figure 24. Selectivity for various dye molecules. MPCM/PAN thin-film composite membranes prepared with 1% aqueous phase concentration.

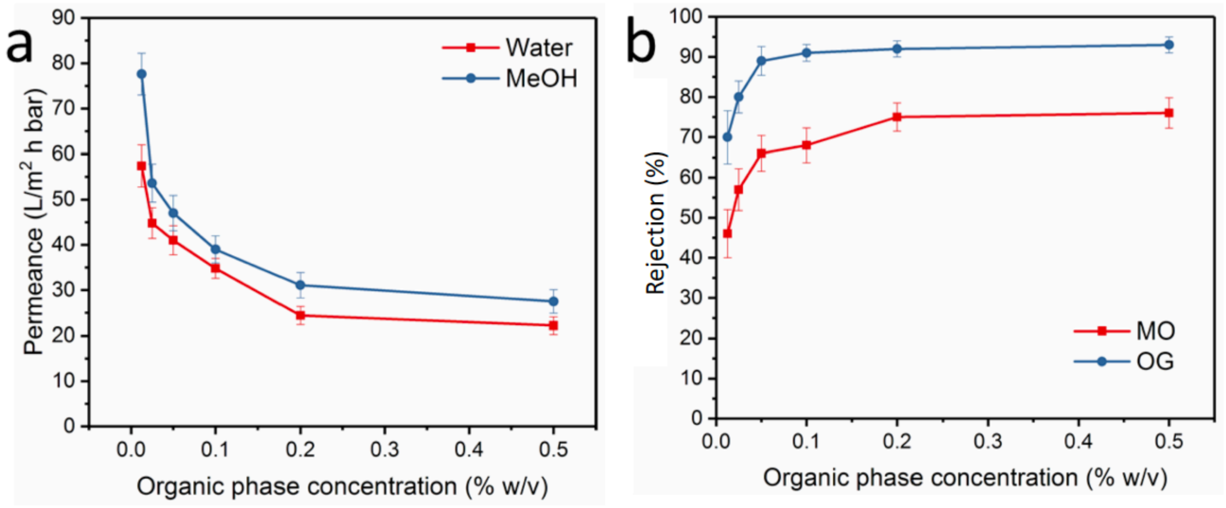

Supplementary Figure 25. **a,** Permeance and **b,** membrane selectivity for various dye molecules by the MPCM/PAN thin-film composite membranes prepared with different organic phase concentration (1% of aqueous phase concentration and reaction time of 10s).

.

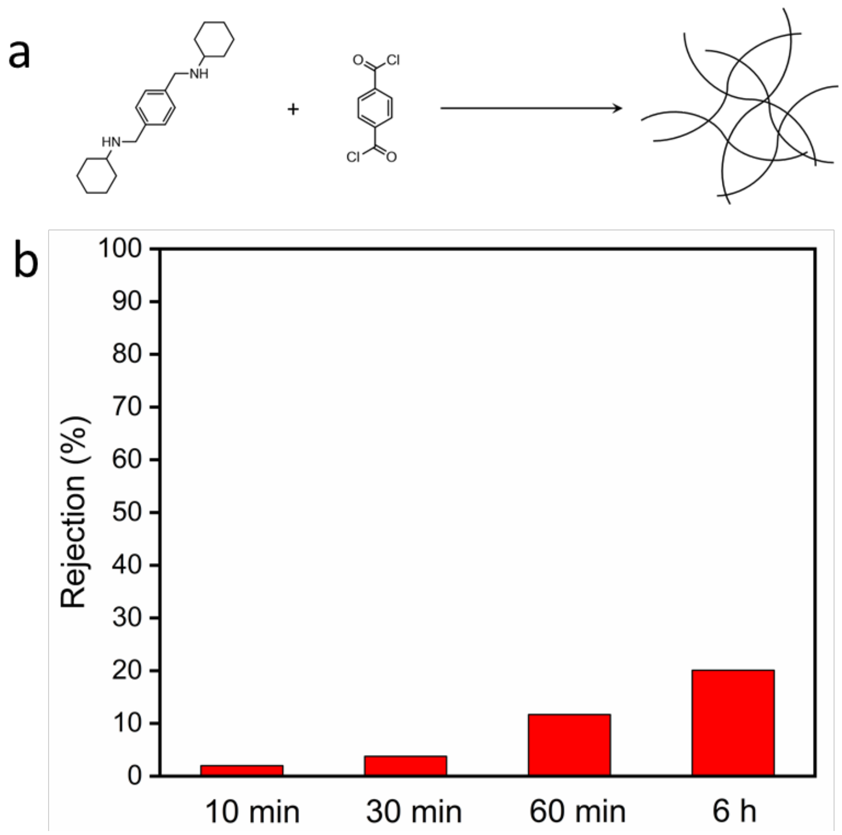

Supplementary Figure 26. a, Interfacial polymerization between trianglamine fragment and TPC. **b,** Membrane selectivity for Congo red by the membranes prepared with different reaction time.

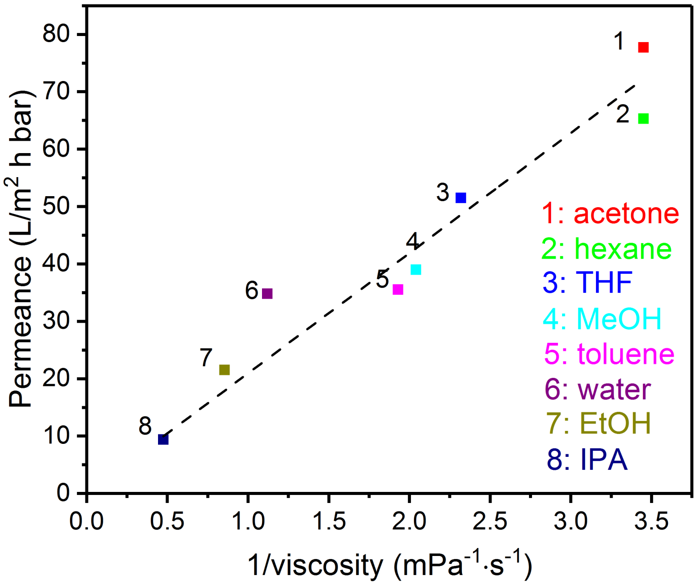

Supplementary Figure 27. Pure solvents through MPCM/PAN thin-film composite membranes as a function of their inverse viscosity.

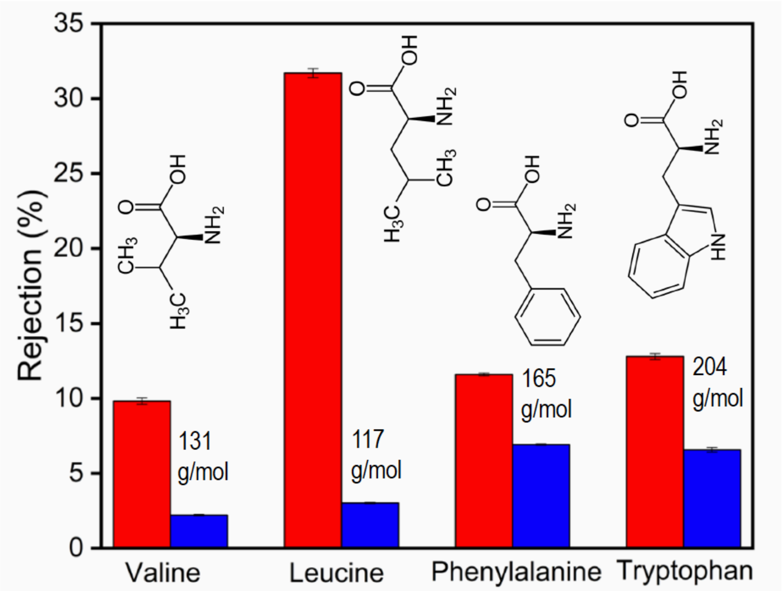

Supplementary Figure 28. Chiral selectivity. MPCM/PAN thin-film composite membranes (Red: Dextrorotary; Blue: Levorotary) rejection of various optically pure amino acids enantiomers.

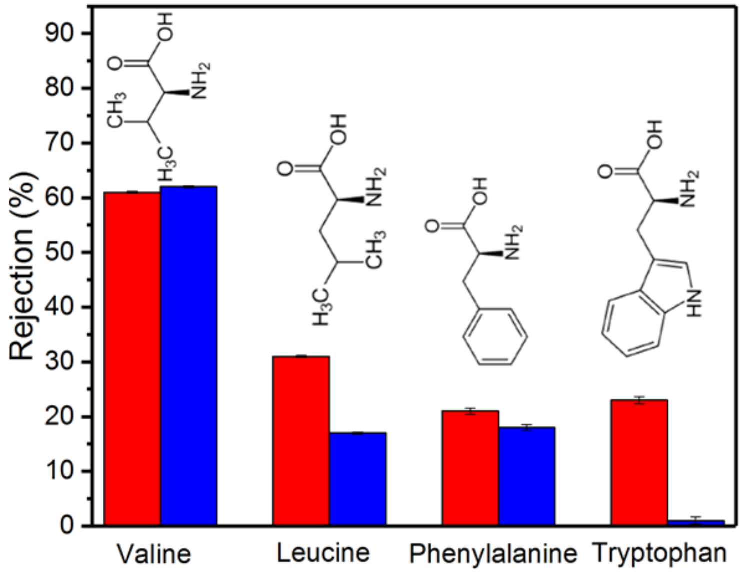

Supplementary Figure 29. Chiral selectivity. MPCM/PAN thin-film composite membranes (Red: Dextrorotary; Blue: Levorotary) rejection of various racemic amino acids.

Supporting molecular modeling

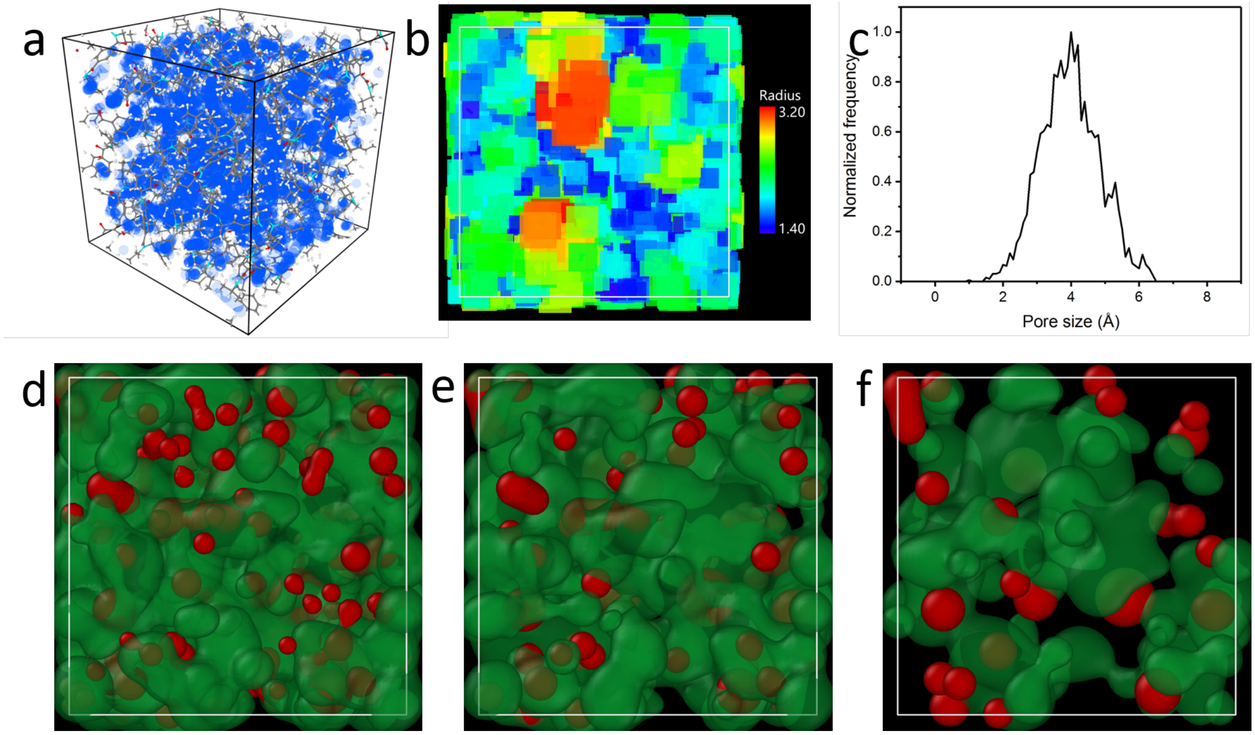

Supplementary Figure 30. Molecular modeling of membrane prepared from TPC and trianglamine fragment, with dimension of 34.78Å × 34.78Å× 34.78Å and accessible surface at probe radius of 1 Å marked blue. **a,** Three-dimensional view of an amorphous cell of the membrane. **b,** Voids distribution with size distinguished by color. **c,** Simulated pore size distributions of the membrane. Interconnected (green) and isolated (red) voids space considering probes of **d,** 0.85 Å, **e,** 1.2 Å, and **f,** 1.55 Å radius, respectively.

Supplementary Table 1. Elements composition of PAN, MPCM/PAN thin-film composite membrane; Freestanding MPCM nanofilm and pristine trianglamine.

	C /%	O /%	N /%
PAN	79.4	0.8	19.7
MPCM/PAN thin-film composite membrane	81.4	7.1	10.2
Freestanding MPCM nanofilm	83.4	9.3	7.3
Trianglamine	89.3	1.6	9.1

Supplementary Table 2. The chemical structures of the dyes used for the molecular separation experiments.

Name	MW (g/mol)	structure	Net charge	Dimension (Å)
Methyl red	291.3	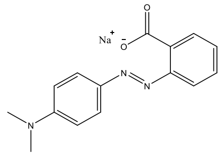	-1	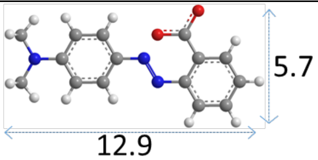
Nile red	318.4	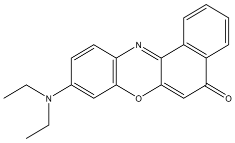	0	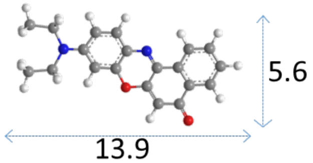
Methylene blue	319.9	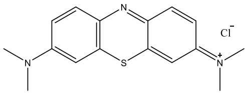	+1	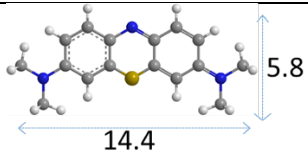
Methyl orange	327.3	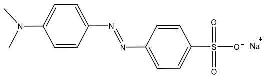	-1	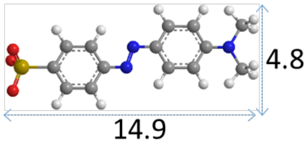
Safranine O	350.8	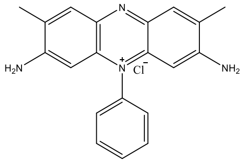	+1	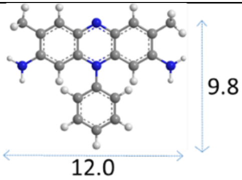
Rhodamine B base	442.6	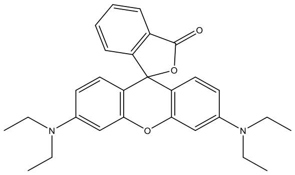	0	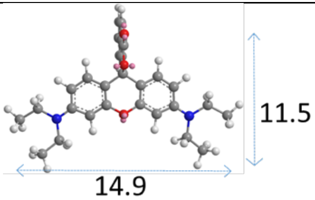
Orange G	452.4	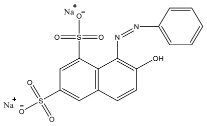	-2	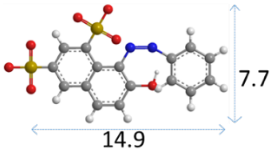
Acid fuchsine	585	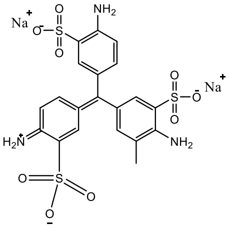	-2	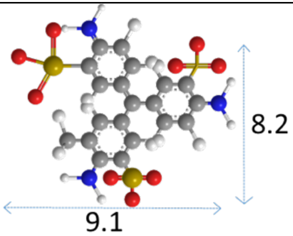
Congo red	696.7	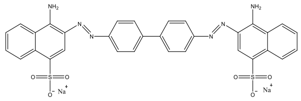	-2	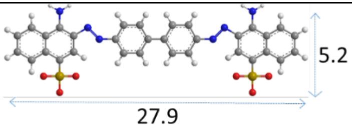

Supplementary Table 3. Summary of the performance of MPCM with the state-of-the-art OSN membranes.

	Membrane	Name	Permeance (L m^{-2} h^{-1} bar^{-1})				rejection	Ref.
			MeOH	acetone	THF	toluene		
Commercial membranes	Polyimide	DuraMem® 150	0.48	-	0.10	-	97% (CV/MeOH)	6, 7, 8
	Polyimide	DuraMem® 500	-	-	-	-	95% (AF/ACN)	9
	Polyimide	PuraMem® 420	-	-	-	-	98% (AF/ACN)	
	Polyimide	Starmem® 122	-	-	-	0.6	87% (PS/toluene)	10
	Polyimide	Starmem® 240	-	-	-	0.7	90% (PS/toluene)	
	Polyamide	Desal-DL	-	-	-	2.6	-	
	Polyamide	Desal-DK	-	-	-	2.6	-	
The state-of-the-art membranes	Polyarylate thin film (XP84 support)	PAR-BHPF	8.0	8.4	4.0	0.3	97% (CV/MeOH)	11
		PAR-TTSBI	6.0	7.0	4.0	2.5	99% (CV/MeOH)	
		PAR-DHAQ	0.6	0.2	0.04	0.01	98% (CV/MeOH)	
		PAR-RES	0.6	0.4	0.04	0.04	100% (CV/MeOH)	
	Polyamide thin film composite membranes (XP84 support)	MPD-4%-1min-ACT	13.25	12.74	1.74	0.18	97.7% (MO/MeOH)	8
		MPD-3%-1min	7.71	-	-	-	98.9% (MO/MeOH)	
		MPD-3%-1min-ACT	19.11	32.9	-	-	95.5% (MO/MeOH)	
		MPD-0.1%-10min-ACT	9.55	11.3	1.3	0.1	96.8% (MO/MeOH)	
	Polyamide thin film composite membranes (alumina support)	MPD-4%-1min-ACT	34.12	31.16	6.02	1.31	98.8% (MO/MeOH)	
		MPD-3%-1min	13.73	19.34	2.59	0.08	98.9%(MO/MeOH)	
		MPD-3%-1min-ACT	52.22	49.68	18.11	3.45	98.9%(MO/MeOH)	
		MPD-0.1%-10min-ACT	12.21	-	-	-	99.7%(MO/MeOH)	
	Conjugated microporous polymers membranes	p-CMP	22.5	-	-	-	99%(PPh-IX/EtOH)	12
		m-CMP	16.4	-	-	-	95%(PPh-IX/EtOH)	
	β-CD thin films on PAN support	β-CD-0.1	0.2	-	-	-	92% (MO/MeOH)	13
		β-CD-0.5	1.8	-	-	-	95% (MO/MeOH)	
		β-CD-1.0	5.8	-	3.2	1.6	93% (MO/MeOH)	
		β-CD-1.5	6.1	-	-	-	94% (MO/MeOH)	
		β-CD-2.0	9.6				91% (MO/MeOH)	
	CD/TMC membranes on PI support	α-CD/TMC	-	-	-	-	98.8% (MO/EtOH)	14
		b-CD/TMC	4.9	-	-	-	95.7% (MO/EtOH)	
		r-CD/TMC	-	-	-		94.6% (MO/EtOH)	
	Polyamide-CD/PAN membranes	Polyamide-CD (0.64)	17.6	24.6	17	19.8	88% (MO/MeOH)	15
		Polyamide-CD (1.28)	2.45	-	-		92% (MO/MeOH)	
	MPCM/PAN thin-film composite membranes	MPCM/PAN(2%, 10s)	22	-	-	-	96% (OG/MeOH)	This work
		MPCM/PAN(2%, 10min)	9.3	-	-	-	98% (OG/MeOH)	
		MPCM/PAN(1%, 10s)	39	77.7	51.5	35.5	91% (OG/MeOH)	
		MPCM/PAN(1%, 10min)	21.3	-	-	-	93% (OG/MeOH)	

Supplementary Table 4. Hansen solubility parameter (δ) and property of solvent used for nanofiltration experiments.

Solvents	Molar volume (V_m)(cm^3 mol^{-1})	d_m (nm)*	Viscosity **(10^{-3} Pa s)	Hansen solubility parameter (δ) ($MPa^{1/2}$)***			
				δ_d	δ_p	δ_h	δ
Water	18.0	0.38	0.89	15.6	16	42.3	47.8
Methanol	40.7	0.51	0.49	15.1	12.3	22.3	29.7
Ethanol	58.5	0.57	1.17	15.8	8.8	19.4	26.6
Isopropanol	76.8	0.62	2.1	15.8	6.1	16.4	23.5
Acetone	73.9	0.62	0.29	15.5	10.4	7.0	21.0
Hexane	131.6	0.75	0.29	14.9	0	0	14.9
Tetrahydrofuran	81.7	0.62	0.43	16.8	5.7	8.0	19.4
Toluene	106.8	0.7	0.52	18.0	1.4	2.0	18.2

*The molar diameter (d_m) was calculated from reference[6] using molar volume (V_m) of the solvent molecule from: $m = 2 \times (3V_m / 4\pi$ N_A $)1/3$; where N_A is the Avogardo's number. **Viscosity taken from reference.[4, 8] ***δ_d = solubility parameter due to dispersion forces, δ_p = solubility parameter due to dipole forces, and δ_h = solubility parameter due to hydrogen bonding (or in general due to donor-acceptor interactions). See reference (Hansen Solubility Parameter Handbook).

Supplementary References

1 Abbott, L., Hart, K. & Colina, C. Polymatic: a generalized simulated polymerization algorithm for amorphous polymers. *Theor. Chem. Acc.* **132**, 1–19 (2013)

2 Abbott, L. & Colina, C. Polymatic: A Simulated Polymerization Algorithm (2013); https://nanohub.org/resources/17278

3 Abbott, L., Hart, K. & Colina, C. Polymatic: a generalized simulated polymerization algorithm for amorphous polymers. *Theor. Chem. Acc.* **132**, 1–19 (2013).

4 Plimpton, S. Fast parallel algorithms for short-range molecular dynamics. *J. Comput. Phys.* **117**, 1–19 (1995).

5 Willems, T. F. et al. Algorithms and tools for high-throughput geometry-based analysis of crystalline porous materials. *Micropor. Mesopor. Mater.* **149**, 134–141 (2012).

6 Marchetti, P., Solomon, M. F. J., Szekely, G. & Livingston, A. G. Molecular Separation with Organic Solvent Nanofiltration: A Critical Review. *Chem. Rev.* **114**, 10735-10806, (2014).

7 Solomon, M. F. J., Bhole, Y. & Livingston, A. G. High flux membranes for organic solvent nanofiltration (OSN)-Interfacial polymerization with solvent activation. *J. Membr. Sci.* **423**, 371-382, (2012).

8 Karan, S., Jiang, Z. W. & Livingston, A. G. Sub-10 nm polyamide nanofilms with ultrafast solvent transport for molecular separation. *Science* **348**, 1347-1351, (2015).

9 Gorgojo, P. *et al.* Ultrathin Polymer Films with Intrinsic Microporosity: Anomalous Solvent Permeation and High Flux Membranes. *Adv. Funct. Mater.* **24**, 4729-4737, (2014).

10 Othman, R., Mohammad, A. W., Ismail, M. & Salimon, J. Application of polymeric solvent resistant nanofiltration membranes for biodiesel production. *J. Membr. Sci.***348**, 287-297, (2010).

11 Jimenez-Solomon, M. F., Song, Q. L., Jelfs, K. E., Munoz-Ibanez, M. & Livingston, A. G. Polymer nanofilms with enhanced microporosity by interfacial polymerization. *Nature Mater.* **15**, 760-+, (2016).

12 Liang, B. et al. Microporous membranes comprising conjugated polymers with rigid backbones enable ultrafast organic-solvent nanofiltration. *Nat. Chem.* **10**, 961-967, (2018).

13 Villalobos, L. F., Huang, T. F. & Peinemann, K. V. Cyclodextrin Films with Fast Solvent Transport and Shape-Selective Permeability. *Adv. Mater.* **29**, 7, (2017).

14 Liu, J. T., Hua, D., Zhang, Y., Japip, S. & Chung, T. S. Precise Molecular Sieving Architectures with Janus Pathways for Both Polar and Nonpolar Molecules. *Adv. Mater.* **30**, 7, (2018).

15 Huang, T., Puspasari, T., Nunes, S. P. & Peinemann, K. V. Ultrathin 2D-Layered Cyclodextrin Membranes for High-Performance Organic Solvent Nanofiltration. *Adv. Funct. Mater.* **30**, 1906797 (2020).
